# IL-23R Signaling Plays No Role in Myocardial Infarction

**DOI:** 10.1038/s41598-018-35188-8

**Published:** 2018-11-20

**Authors:** Erika Engelowski, Nastaran Fazel Modares, Simone Gorressen, Pascal Bouvain, Dominik Semmler, Christina Alter, Zhaoping Ding, Ulrich Flögel, Jürgen Schrader, Haifeng Xu, Philipp A. Lang, Jens Fischer, Doreen M. Floss, Jürgen Scheller

**Affiliations:** 1 Institute of Biochemistry and Molecular Biology II, Medical Faculty, Heinrich-Heine University, 40225 Düsseldorf, Germany; 2 Institute of Pharmacology and Clinical Pharmacology, Medical Faculty, Heinrich-Heine University, 40225 Düsseldorf, Germany; 3 Institute for Molecular Cardiology, Medical Faculty, Heinrich-Heine University, 40225 Düsseldorf, Germany; 4 Institute of Molecular Medicine II, Medical Faculty, Heinrich-Heine-University, Düsseldorf, Germany

## Abstract

Ischemic heart diseases are the most frequent diseases in the western world. Apart from Interleukin (IL-)1, inflammatory therapeutic targets in the clinic are still missing. Interestingly, opposing roles of the pro-inflammatory cytokine IL-23 have been described in cardiac ischemia in mice. IL-23 is a composite cytokine consisting of p19 and p40 which binds to IL-23R and IL-12Rβ1 to initiate signal transduction characterized by activation of the Jak/STAT, PI3K and Ras/Raf/MAPK pathways. Here, we generate IL-23R-Y416FΔICD signaling deficient mice and challenged these mice in close- and open-chest left anterior descending coronary arteria ischemia/reperfusion experiments. Our experiments showed only minimal changes in all assayed parameters in IL-23R signaling deficient mice compared to wild-type mice in ischemia and for up to four weeks of reperfusion, including ejection fraction, endsystolic volume, enddiastolic volume, infarct size, gene regulation and α smooth muscle actin (αSMA) and Hyaluronic acid (HA) protein expression. Moreover, injection of IL-23 in wild-type mice after LAD ischemia/reperfusion had also no influence on the outcome of the healing phase. Our data showed that IL-23R deficiency has no effects in myocardial I/R.

## Introduction

The immune system and their cytokines contribute to the healing processes after myocardial infarction^1^. Apart from Interleukin (IL-)1, inflammatory therapeutic targets in the clinic are still missing^2^. It is, however, in most cases still unclear to which extent different cytokines are involved in positive or negative remodeling of the heart after myocardial infarction in preclinical models of acute heart failure. Some cytokines having even positive and negative effects depending on the time after myocardial infarction, as exemplified by Interleukin (IL-)6, which is protective in the early phase but becomes detrimental if chronically produced^3^. For the closely related IL-23 different outcomes after myocardial infarction have been described in mice. Savvatis *et al*. reported a protective role of IL-23 signaling in a murine model of permanent occlusion of the left anterior descending artery (LAD). They showed that IL-23p19 deficient mice had an overall reduced survival rate, higher expression of inflammatory cytokines and infiltration of inflammatory cells accompanied by impaired healing process^4^. In opposite, Yan *et al*. showed that infarct size in IL-23p19 deficient mice was reduced and overall survival was increased after myocardial infarction (permanent ligation) compared to wild-type mice^5^. This finding was supported by studies in rats. Here, rats injected with recombinant IL-23 after acute ischemia/reperfusion had increased infarct sizes and upregulation of inflammatory cytokines, whereas blockade of IL-23 by a neutralizing anti-IL-23 antibody resulted in reduced infarct sizes and less apoptotic cells^6^.

The reason for these different phenotypes remain unclear but might be related to the complex biology of IL-12-type cytokines. The IL-12 family of cytokines is comprised of IL-12, IL-23, IL-27, IL-35 and IL-39^7^. These cytokines consist of soluble heterodimers formed by α and β subunits. The cytokine α chain IL-23p19 is structurally related to IL-6 and forms complexes with the soluble receptor subunit IL-12p40 (β chain). IL-39 (also named IL-X) is also composed of IL-23p19 but forms a complex with EBI3 which is also engaged with p28 in IL-27^8^. IL-23 signals via the heterodimeric receptor complex IL-23R:IL-12Rβ1, in which the IL-23R is regarded as the only signal-transducing component of the IL-23 receptor complex^9^. The receptor complex of IL-39 is not known, however, combinations of IL-23R:gp130 were recently proposed^8,10^. IL-23 activates mainly STAT3 but also STAT1, 4 and 5^11^. Some of the pro-inflammatory functions of IL-23 are related to the induction of terminal differentiation and proliferation of IL-17-producing CD4^+^ T helper (T_H_17) cells^12^. Consequently, IL-23 (p19) or the IL-23 receptor (IL-23R) deficient mice are resistant to autoimmune and inflammatory disorders, such as experimental autoimmune encephalomyelitis^13^, collagen-induced arthritis^14^ and inflammatory bowel disease (IBD)^15,16^. A number of cell types express IL-23 receptor chains, including CD4^+^ T cells of the T_H_17 lineage, γδ T cells, macrophages, dendritic cells, and innate lymphoid cells^17,18^.

Using site-directed mutagenesis and deletion variants of the murine and human IL-23R, we have previously defined canonical and non-canonical STAT and MAPK binding/activation sites within the murine IL-23R. The membrane-proximal Y416 in murine IL-23R is involved in the activation of PI3K/AKT and the MAPK pathway upon stimulation with IL-23^19^.

Here we generated a novel IL-23R-Y416FΔICD signaling deficient mice and challenged these mice in myocardial ischemia/reperfusion. These mice are characterized by the amino acid exchange Y416F and deletion of most of the intracellular domain beginning at amino acid residue 433 to abrogate canonical and non-canonical STAT binding/activating sites of the intracellular domain of the IL-23R. Surprisingly, these mice revealed that IL-23R signaling plays no role in the healing phase of myocardial infarction after ischemia/reperfusion.

## Results

### Generation of IL-23R signaling deficient mice

**Figure 1:**
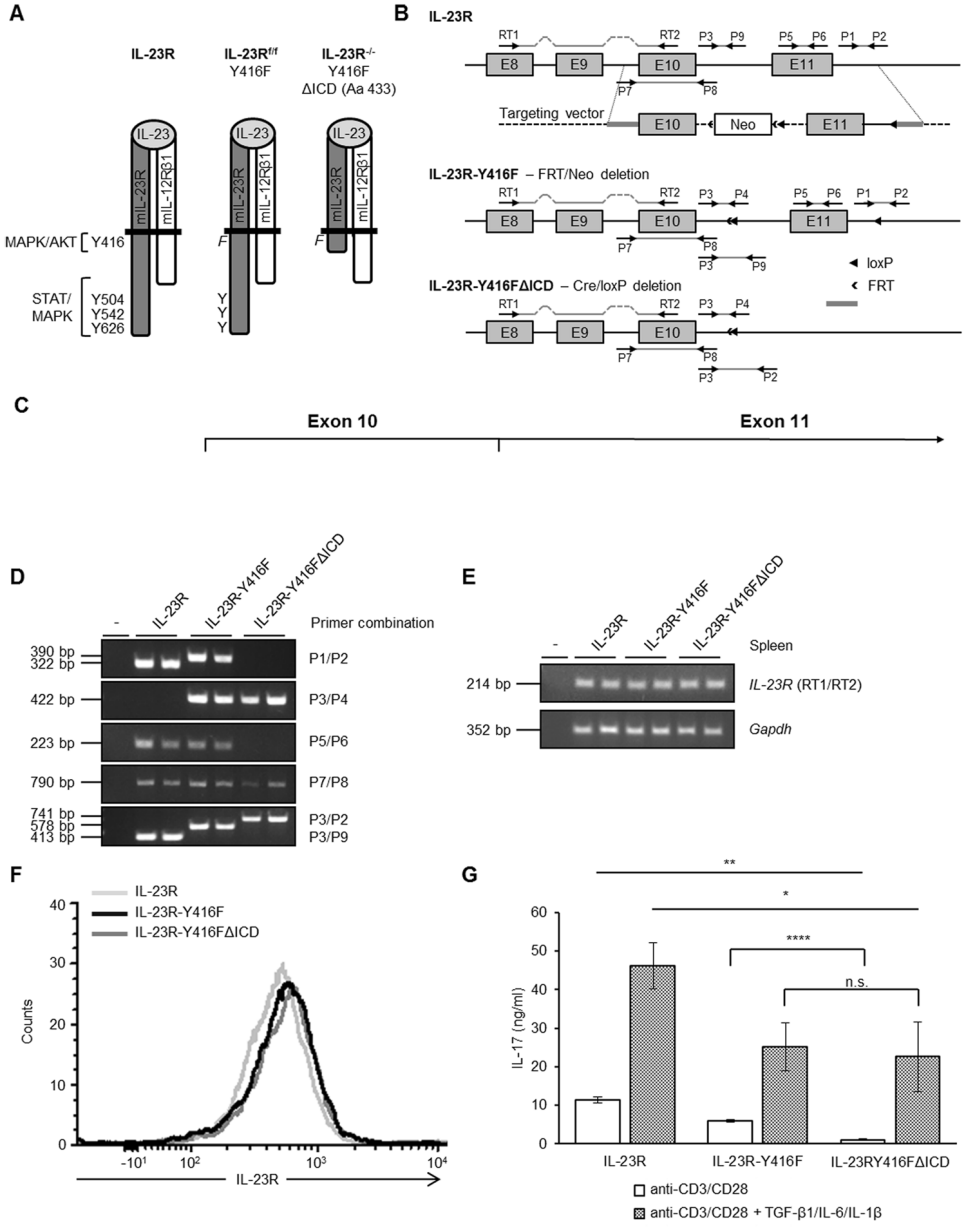
Generation of IL-23 signaling deficient mice. (**A**) Schematic illustration of IL-23 receptor chains IL-23R and IL-12Rβ1 in complex with IL-23. Indicated are the canonical tyrosine residues and the non-canonical region for binding/activation of AKT, MAPK and STAT signaling. In IL-23R-Y416F mice tyrosine 416 is mutated into phenylalanine and in IL-23R-Y416FΔICD signaling deficient mice Y416F is combined with a deletion of the IL-23R from amino acid residue 432. (**B**) Targeting strategy for the generation of floxed IL-23-Y416F and IL-23R-Y416FΔICD signaling deficient mice. The arrows indicate the locations of primers used for genomic PCR and RT-PCR. (**C**) Partial amino acid sequence of IL-23R, IL-23R-Y416F and IL-23R-Y416FΔICD mice. (**D**) PCR to characterize genomic organization of IL-23R, IL-23R-Y416F and IL-23R-Y416FΔICD in mice. Primer combinations are indicated. (**E**) RT-PCR on RNA from spleen tissue of IL-23R, IL-23R-Y416F and IL-23R-Y416FΔICD mice. GAPDH served as control. (**F**) IL-23R cell surface expression of spleen cells from IL-23R (light gray), IL-23R-Y416F (black) and IL-23R-Y416FΔICD (dark gray) mice 5 days after stimulation with anti-CD3, anti-CD28, TGF-β1, IL-6 and IL-1 by flow cytometry. (**G**) Splenocytes were incubated for 5 days with anti-CD3 and anti-CD28, in the absence (white bars) or presence of TGF-β1, IL-6 and mouse IL-1β (black dotted bars). Supernatants were collected and analyzed by ELISA for IL-17. Results are mean ± S.D. of three replicates. Significance of difference (two-tailed Student t test): *p < 0.05, **p < 0.01, ****p < 0.0001.

In order to analyze the role of IL-23 signaling in left anterior descending coronary arteria (LAD) ischemia/reperfusion, we generated mice with either defective AKT signaling or complete loss of signaling of IL-23. The intracellular domain of the IL-23R is coded by exon 10 and exon 11. The first tyrosine in the intracellular domain is responsible for AKT/ERK signaling and coded by exon 10. Moreover, canonical and non-canonical STAT and ERK signaling is mediated by amino acids coded in exon 11 (Fig. 1A)^19^. First of all, we introduced a point mutation in exon 10, resulting in the amino acid exchange Y416F abrogating AKT signaling^19^ and exon 11 was flanked by loxP sites. As a consequence floxed IL-23R-Y416F mice are defective for AKT signaling. Cre-mediated recombination resulted in deletion of exon 11 and abrogated STAT and ERK signaling. These mice are referred to as IL-23R-Y416FΔICD signaling deficient mice (Fig. 1B). Deletion of exon 11 led to a shortened intracellular domain with 14 alternative amino acids coded by the former intron 11 until translation is stopped by a new stop codon (Fig. 1C).

Here, IL-23R-Y416F mice were crossed with a CRE-deleter, which resulted in deletion of IL-23R exon 11 in the germline. Introduction of Y416F and the loxP sites in IL-23R-Y416F mice and deletion of exon 11 in IL-23R-Y416FΔICD signaling deficient mice was confirmed by genomic PCR (Fig. 1B,D,E). Finally, we verified expression of wild-type-IL-23R, IL-23R-Y416F and IL-23RY416FΔICD by flow cytometry on spleen cells of IL-23R, IL-23R-Y416F and IL-23R-Y416FΔICD signaling deficient mice (Fig. 1F), demonstrating that mutation and/or deletion of the intracellular domain did not disturb expression and presentation of the mutated and/or truncated IL-23R. Next, spleen cells from wild-type-IL-23R, IL-23R-Y416F and IL-23RY416FΔICD mice were stimulated with anti-CD3 and anti-CD28 mAbs in the presence or absence of TGF-β, IL-6 and IL-1β to induce IL-17 production. IL-23 signaling critically contributes to IL-17 production after CD3/CD28 and/or cytokine stimulation^20^, which was significantly reduced in spleen cells from IL-23R-Y416F and IL-23RY416FΔICD mice as compared to IL-23R (wild-type) mice (Fig. 1G). Moreover, IL-17 is also produced after selective stimulation of CD3/CD28, albeit to a lesser extent. Importantly, in spleen cells from IL-23R-Y416F but to a higher degree from IL-23RY416FΔICD mice IL-17 expression was significantly suppressed (Fig. 1G). Taken together, we generated and characterized a novel IL-23R signaling deficient knock out mouse.

### Abrogation of IL-23 signaling has no effect on myocardial remodeling after closed-chest LAD ischemia/reperfusion

**Figure 2:**
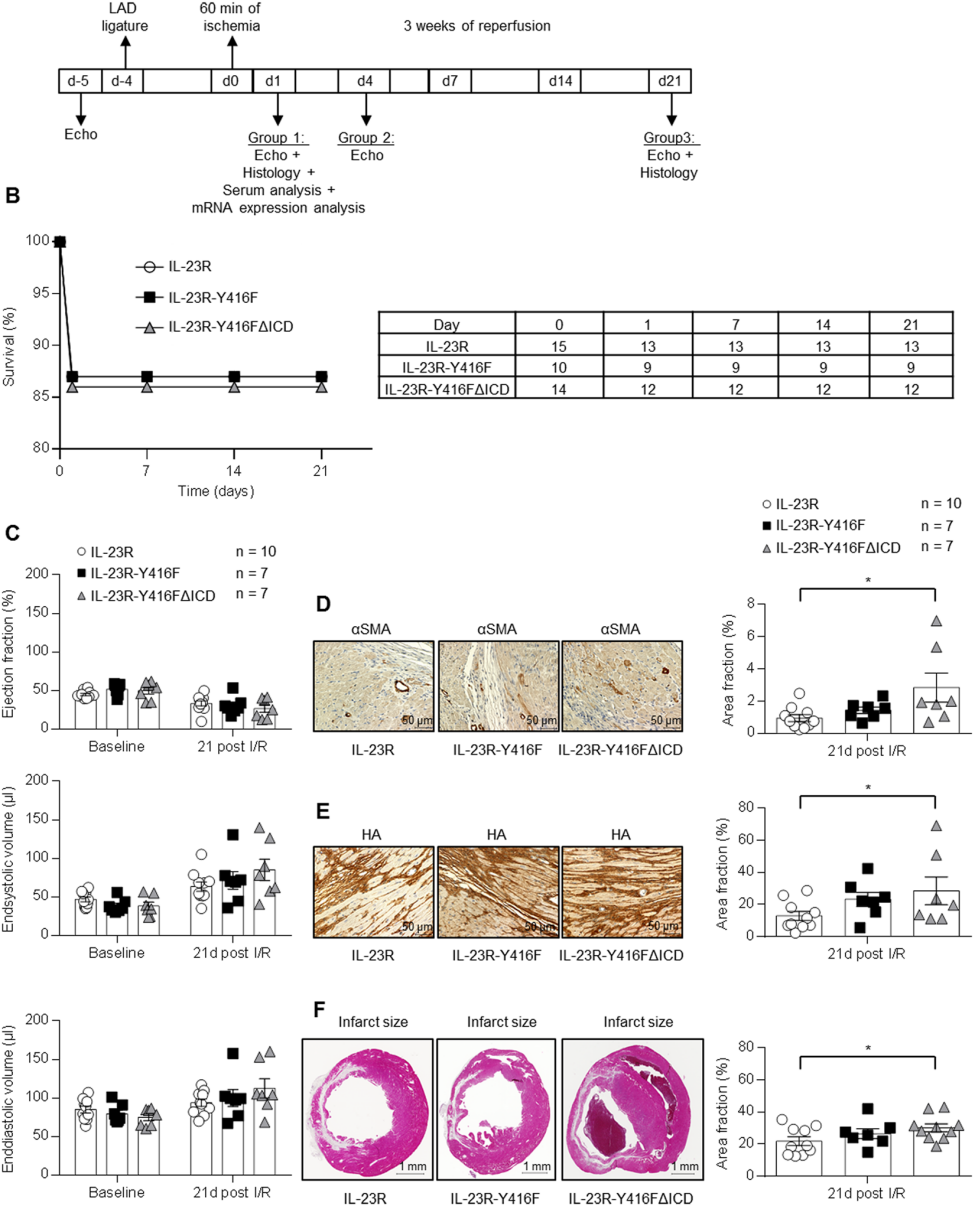
Closed-chest LAD ischemia/reperfusion with 60 min ischemia and 3 weeks reperfusion in IL-23R, IL-23R-Y416F and IL-23R-Y416FΔICD signaling deficient mice. (**A**) Schematic illustration of the closed-chest LAD experiment with IL-23R, IL-23R-Y416F and IL-23R-Y416FΔICD mice. Baseline measurement were performed at day -5, LAD ligature was inserted at day -4, 60 min of ischemia was done at day 0 followed by 3 weeks of reperfusion and analysis at day 1, 4 and 21. (**B**) Survival curve of IL-23R (n = 15), IL-23R-Y416F (n = 10) and IL-23R-Y416FΔICD (n = 14) mice after ischemia/reperfusion. (**C**) Echocardiographic analysis of ejection fraction, endsystolic- and enddiastolic volume before (baseline) and after 3 weeks of reperfusion. Results are mean ± S.E.M. of 7–10 animals/group (IL-23R n = 10; IL-23R-Y416F n = 7; IL-23R-Y416FΔICD n = 7). (**D**) Immunostaining and quantitative analysis of αSMA protein amounts in the left ventricle (LV) 3 weeks of reperfusion; 40x magnification; scale bars, 50 µm. Results are mean ± S.E.M. of 7–10 animals/group (IL-23R n = 10; IL-23R-Y416F n = 7; IL-23R-Y416FΔICD n = 7). (**E**) Immunostaining with HAbP and quantitative analysis of HA amounts in the LV 3 weeks of reperfusion. Representative images of the LV after 3 weeks of reperfusion and quantitative image analysis; 40x magnification; scale bars, 50 µm. Results are mean ± S.E.M. of 7–10 animals/group (IL-23R n = 10; IL-23R-Y416F n = 7; IL-23R-Y416FΔICD n = 7). (**F**) Representative Gomori’s 1-step trichrome staining of heart sections and quantitative analysis of infarct size/LV 3 weeks of reperfusion; scale bars, 1 mm. Results are mean ± S.E.M. of 7–10 animals/group (IL-23R n = 10; IL-23R-Y416F n = 7; IL-23R-Y416FΔICD n = 10). Significance of difference (one-way ANOVA): *p < 0.05.

In a first step, closed-chest LAD ischemia/reperfusion (I/R) was induced in IL-23R (wild-type), IL-23R-Y416F and in IL-23R-Y416FΔICD signaling deficient mice (Fig. 2A). The closed-chest model was choosen because it reflects the symptoms of acute cardiac syndromes and thus mimics the condition of patients who appear after an acute myocardial infarction. LAD ligature was performed for all three genotypes and the chest and skin were closed again. To exclude that preconditioning could influence the ischemia/reperfusion results there was a break of 4 days between LAD operation and induction of ischemia. At postoperative day 4, ischemia for 60 min was induced followed by 3 weeks reperfusion. The heart function in mice was analyzed by echocardiography and the expression of α-SMA and HA was performed by immunostaining. The infarct size was quantified using Gomori’s 1-step trichrome staining. Overall survival of all mouse groups was nearly identical with about 85% until 21 days after LAD ischemia/reperfusion (Fig. 2B). The cardiac function, resulting from the analysis of LV ejection fraction, endsystolic volume and enddiastolic volume, was not statistically significant different between the three mouse strains as determined by echo-analysis at baseline and 3 weeks after I/R (Fig. 2C). However, a trend towards lower ejection fraction and higher endsystolic volume and enddiastolic volume in the IL-23R-Y416FΔICD signaling deficient mice compared to IL-23R mice was apparent. Moreover, we analyzed myocardial wound healing and myofibroblast differentiation after 3 weeks of reperfusion. Importantly, the expression of α smooth muscle actin (αSMA), Hyaluronic acid (HA) and the infarct size demonstrated a significant increase in the IL-23R-Y416FΔICD signaling deficient mice compared to IL-23R mice (Fig. 2D–F). No significant difference was observed in IL-23R-Y416F mice as compared to wild-type mice (Fig. 2D–F).

**Figure 3:**
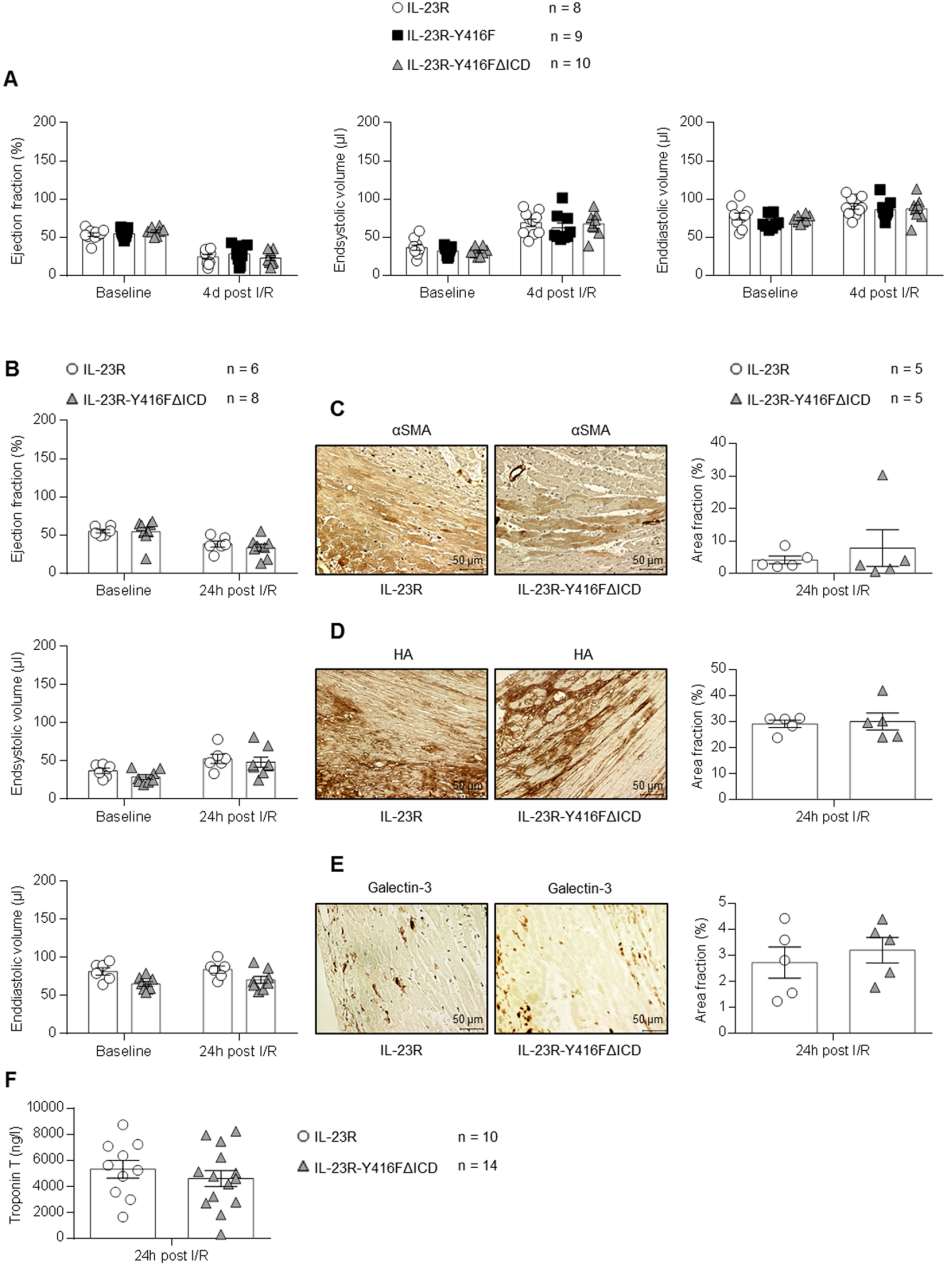
Closed-chest LAD ischemia/reperfusion with 60 min ischemia and 24 h/4d reperfusion in IL-23R and IL-23R-Y416FΔICD signaling deficient mice. (**A**) Echocardiographic analysis of ejection fraction, endsystolic- and enddiastolic volume before (baseline) and after 4 days of reperfusion. Results are mean ± S.E.M. of 8–10 animals/group (IL-23R n = 8; IL-23R-Y416FΔICD n = 10). (**B**) Echocardiographic analysis of ejection fraction, endsystolic- and enddiastolic volume before (baseline) and after 24 h of reperfusion. Results are mean ± S.E.M. of 6–8 animals/group (IL-23R n = 6; IL-23R-Y416FΔICD n = 8). (**C**) Immunostaining and quantitative analysis of αSMA protein amounts in the left ventricle (LV) after 24 h of reperfusion; 40x magnification; scale bars, 50 µm. Results are mean ± S.E.M. of 5 animals/group. (**D**) Immunostaining with HAbP and quantitative analysis of HA amounts in the LV after 3 weeks of reperfusion. Representative images of the LV after 3 weeks of reperfusion and quantitative image analysis; 40x magnification; scale bars, 50 µm. Results are mean ± S.E.M. of 5 animals/group. (**E**) Immunostaining and quantitative analysis of Galectin-3 protein amounts in the left ventricle (LV) after 24 h of reperfusion; 40x magnification; scale bars, 50 µm. Results are mean ± S.E.M. of 5 animals/group. (**F**) Measurement of mouse serum samples for troponin T 24 h after I/R. Results are mean ± S.E.M. of 6–14 animals/group (IL-23R n = 10; IL-23R-Y416F n = 6; IL-23R-Y416FΔICD n = 14).

**Figure 4:**
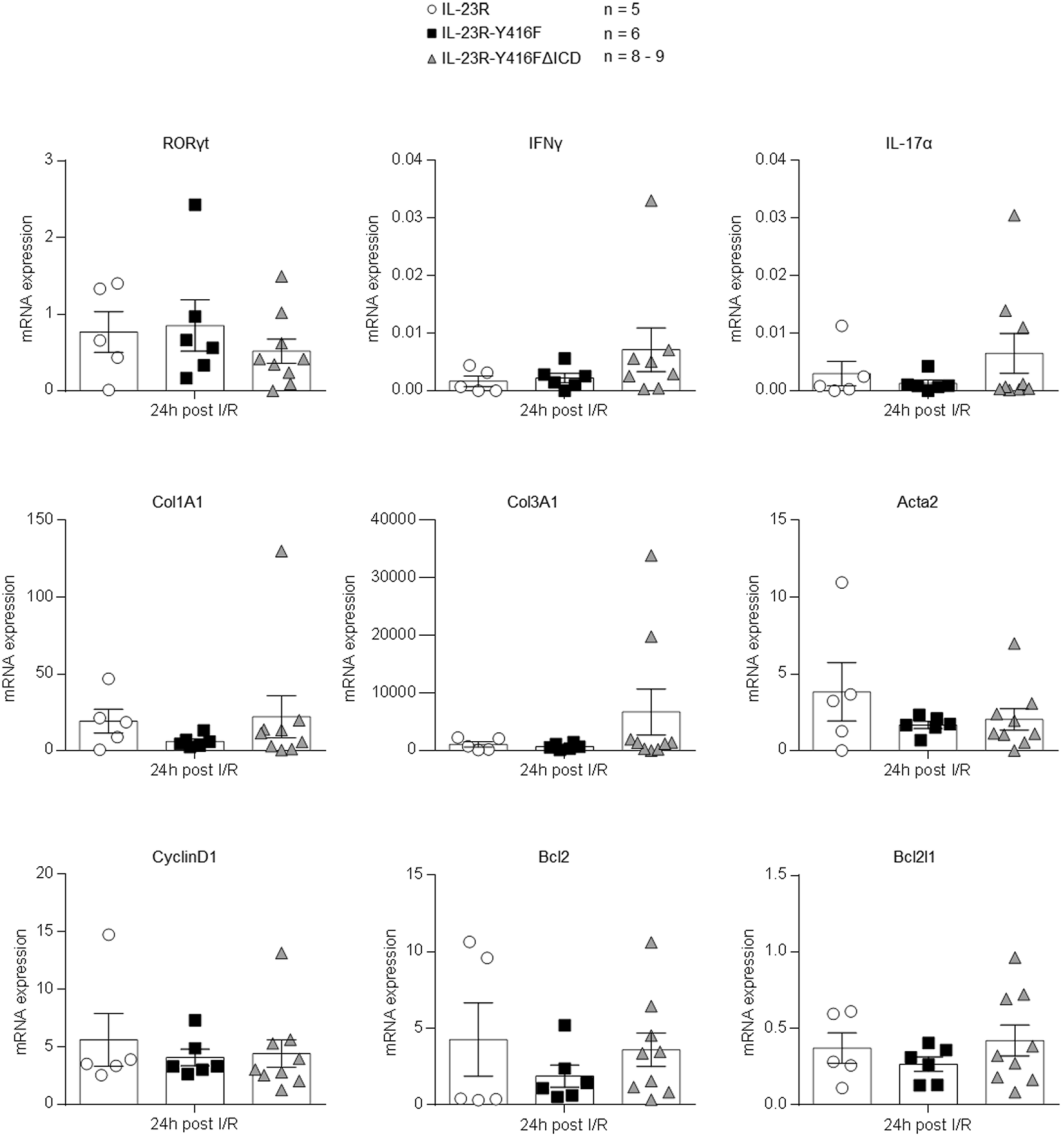
Real-time PCR analysis of gene expression 24 h after LAD ischemia/reperfusion. *RORγt, IFN-γ*, *IL-17α*, *Col1A1, Col3A1, Acta2, Bcl2l1, CyclinD1 and Bcl2* mRNA levels in the LV of IL-23R, IL-23R-Y416F and IL-23R-Y416FΔICD mice after 24 h of reperfusion. qPCR has been performed as described under “Experimental procedures”. Results are mean ± S.E.M. of 5–9 animals/group (IL-23R n = 5; IL-23R-Y416F n = 6; IL-23R-Y416FΔICD n = 8–9).

The parameters for determining the LV cardiac function were also analyzed after 24 h and 4 d of reperfusion. As expected, after 24 h and 4 d of reperfusion no differences in ejection fraction, endsystolic volume and enddiastolic volume were observed (Fig. 3A,B). Next, IL-23R-Y416FΔICD signaling deficient mice and IL-23R mice were sacrificed after ischemia and 24 h of reperfusion and the hearts were stained for αSMA, HA and Galectin-3. Here, expression of both α-SMA and HA was not significantly increased after 24 h in IL-23R-Y416FΔICD signaling deficient mice compared to IL-23R mice, however, a trend for increased αSMA was detected (Fig. 3C,D). Staining of Galectin-3 also showed a trend for increased macrophage infiltration after 24 h of reperfusion in IL-23R-Y416FΔICD (Fig. 3E). To assess the severity of myocardial tissue damage troponin T was measured in the serum of IL-23R-Y416FΔICD signaling deficient mice and IL-23R mice. No differences were observed among the different genotypes after 24 h of reperfusion (Fig. 3F). Finally we analyzed the expression of genes by real-time PCR which are typically regulated early after closed-chest ischemia/reperfusion. Importantly, 24 h after reperfusion neither the inflammatory factors RORγt, IFNγ and IL-17α, nor the cell-cycle and anti-apoptotic factors CyclinD1, Bcl2l1 and Bcl2 or the structural proteins ColA1, Col3A1 and Acta1 (actin) were significantly differential regulated in IL-23R, IL-23R-Y416F and IL-23-Y416FΔICD signaling deficient mice (Fig. 4). Again, only a trend towards increased expression of IFNγ, IL-17α and Col3A1 was observed in IL-23-Y416FΔICD signaling deficient mice as compared to wild-type mice.

Surprisingly, our data indicated that abrogated IL-23 signaling has no effects on the outcome of close-chest LAD ischemia/reperfusion. This finding was not expected, since several groups have previously reported positive and negative effects of IL-23 signaling on permanent acute myocardial infarction in mice^4,5^. Since the infarct sizes in closed-chest models are much smaller than in permanent occlusion of the LAD, we applied the alternative open-chest ischemia/reperfusion model in which the reperfusion can be visually inspected and which should give additional independent information about the role of IL-23 in ischemia/reperfusion.

### Abrogated IL-23 signaling has also no effects on the outcome of open-chest LAD ischemia/reperfusion

**Figure 5:**
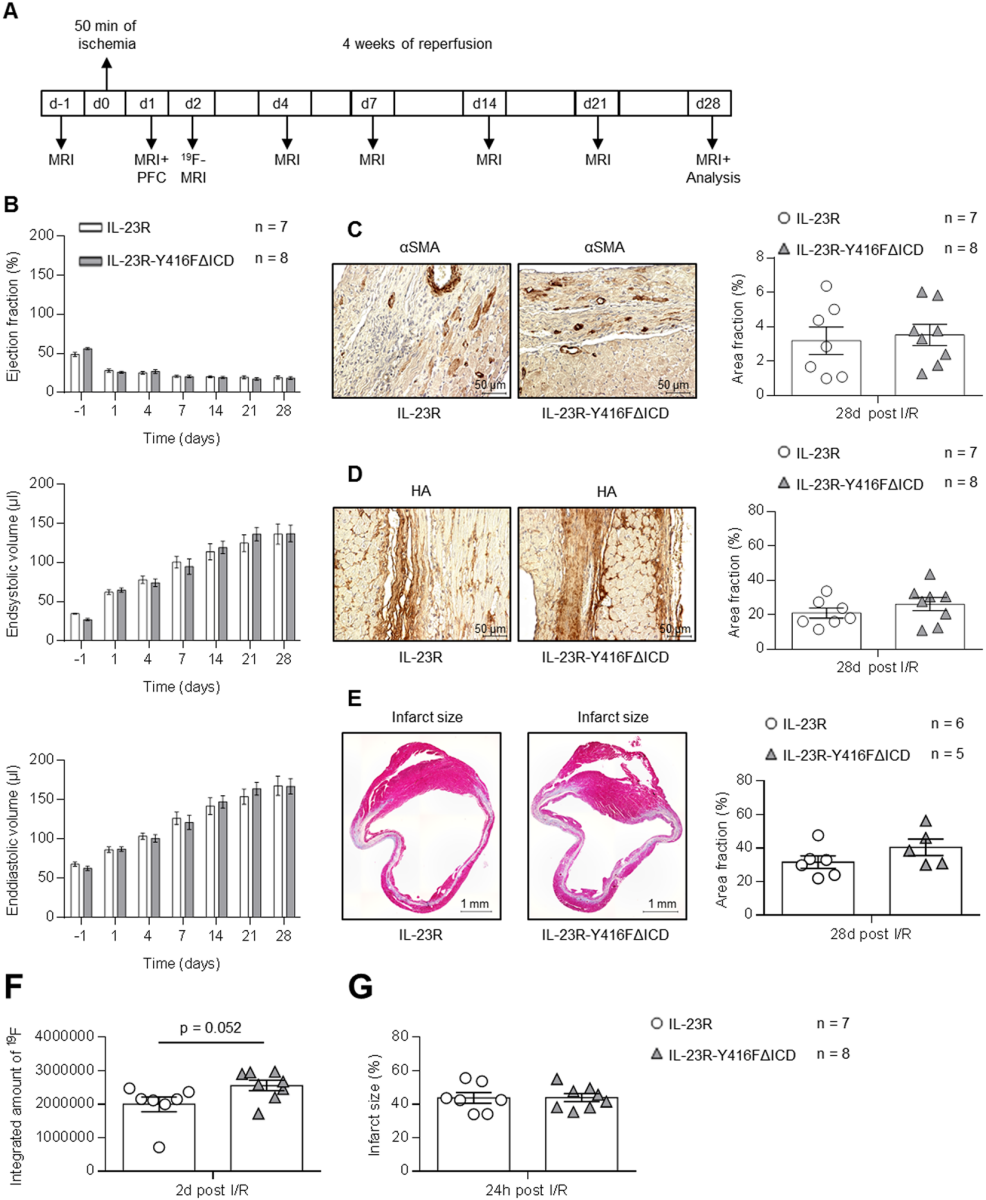
Open-chest LAD ischemia/reperfusion with 50 min ischemia and 4 weeks reperfusion in IL-23R and IL-23R-Y416FΔICD signaling deficient mice. (**A**) Schematic illustration of the open-chest LAD experiment with IL-23R and IL-23R-Y416FΔICD mice. Baseline measurement were performed at day -1, LAD ligature and 50 min of ischemia was done at day 0 followed by 4 weeks of reperfusion and analysis at day 1, 4, 7, 14, 21 and 28. (**B**) MRI analysis of ejection fraction, endsystolic- and enddiastolic volume before (baseline) and after 24 h, 4 d, 7 d, 14 d, 21 d and 28 d of reperfusion. Results are mean ± S.E.M. of 7–8 animals/group (IL-23R n = 7; IL-23R-Y416FΔICD n = 8; baseline: IL-23R n = 3; IL-23R-Y416FΔICD n = 4). (**C**) Immunostaining and quantitative analysis of αSMA protein in the left ventricle (LV) after 28 days of reperfusion; 40x magnification; scale bars, 50 µm. Results are mean ± S.E.M. of 7–8 animals/group (IL-23R n = 7; IL-23R-Y416FΔICD n = 8). (**D**) Immunostaining with HAbP and quantitative analysis of HA amounts in the LV after 3 weeks of reperfusion. Representative images of the LV after 3 weeks of reperfusion and quantitative image analysis; 40x magnification; scale bars, 50 µm Results are mean ± S.E.M. of 7–8 animals/group (IL-23R n = 7; IL-23R-Y416FΔICD n = 8). (**E**) Representative Gomori’s 1-step trichrome staining of heart sections and quantitative analysis of infarct size/LV after 28 days of reperfusion; scale bars, 1 mm. Results are mean ± S.E.M. of 5–6 animals/group (IL-23R n = 6; IL-23R-Y416FΔICD n = 5). (**F**) Calculated amounts of ^19^F as indicator of macrophages infiltration after 24 h of reperfusion by magnetic resonance imaging as described in “Experimental procedures”. (**G**) Ischemic areas of hearts after 24 h of reperfusion was visualized and calculated by magnetic resonance imaging as described in “Experimental procedures”.

Open-chest model reflects the clinical cardiac surgical procedures. Compared to closed-chest model, the open-chest model is accompanied by stronger inflammatory reactions which influence the myocardial remodeling and results in a much larger infarct size. With this model it is possible to study the myocardial injury not only as a consequence of myocardial ischemia/reperfusion but also of the surgical opening of the chest. Furthermore, the open-chest model allows a better control over reperfusion. Consequently, we challenged the IL-23R (wild-type) and IL-23R-Y416FΔICD signaling deficient mice in open-chest ischemia/reperfusion experiments with 50 min of ischemia and 4 weeks of reperfusion (Fig. 5A). Here the LAD was occluded immediately after the ligature and reperfusion was initiated after 50 min. The left ventricle function was analyzed by magnetic resonance imaging (MRI) because of the higher spatial and temporal resolution. Ejection fraction, endsystolic volume and enddiastolic volume at baseline and after 24 h, 4 d, 7 d, 14 d, 21 d and 28 d of reperfusion revealed no differences between IL-23R-wild-type and IL-23R-Y416FΔICD signaling deficient mice (Fig. 5B). After 28 d of open-chest I/R the mice were sacrificed and the hearts were taken for histological analysis. Staining of αSMA and HA in IL-23R-wild-type and IL-23R-Y416FΔICD mice revealed no significant differences (Fig. 5C and D). Also the quantification of myocardial infarct sizes showed no differences in both genotypes (Fig. 5E).

Before the mice were sacrificed, we performed MRI analysis after the injection of perfluorcarbon (F19) particles to quantify the phagocyte infiltration into the heart after 2 d of ischemia/reperfusion. As shown in Fig. 5F, a strong trend (p = 0.052) of increased F19 accumulation as indicator of infiltrating inflammatory phagocytes in the hearts of IL-23R-Y416FΔICD signaling deficient mice compared to IL-23R-wild-type mice was observed. Moreover, the interstitial space was determined 24 h after reperfusion by MRI and the extent of myocardial infarction was assessed. Here, we did not observe any differences in the infarct size in the hearts of IL-23R-Y416FΔICD signaling deficient mice compared to IL-23R-wild-type mice (Fig. 5G).

### Therapeutic treatment with IL-23 has no effect on the outcome of open-chest ischemia/reperfusion

**Figure 6:**
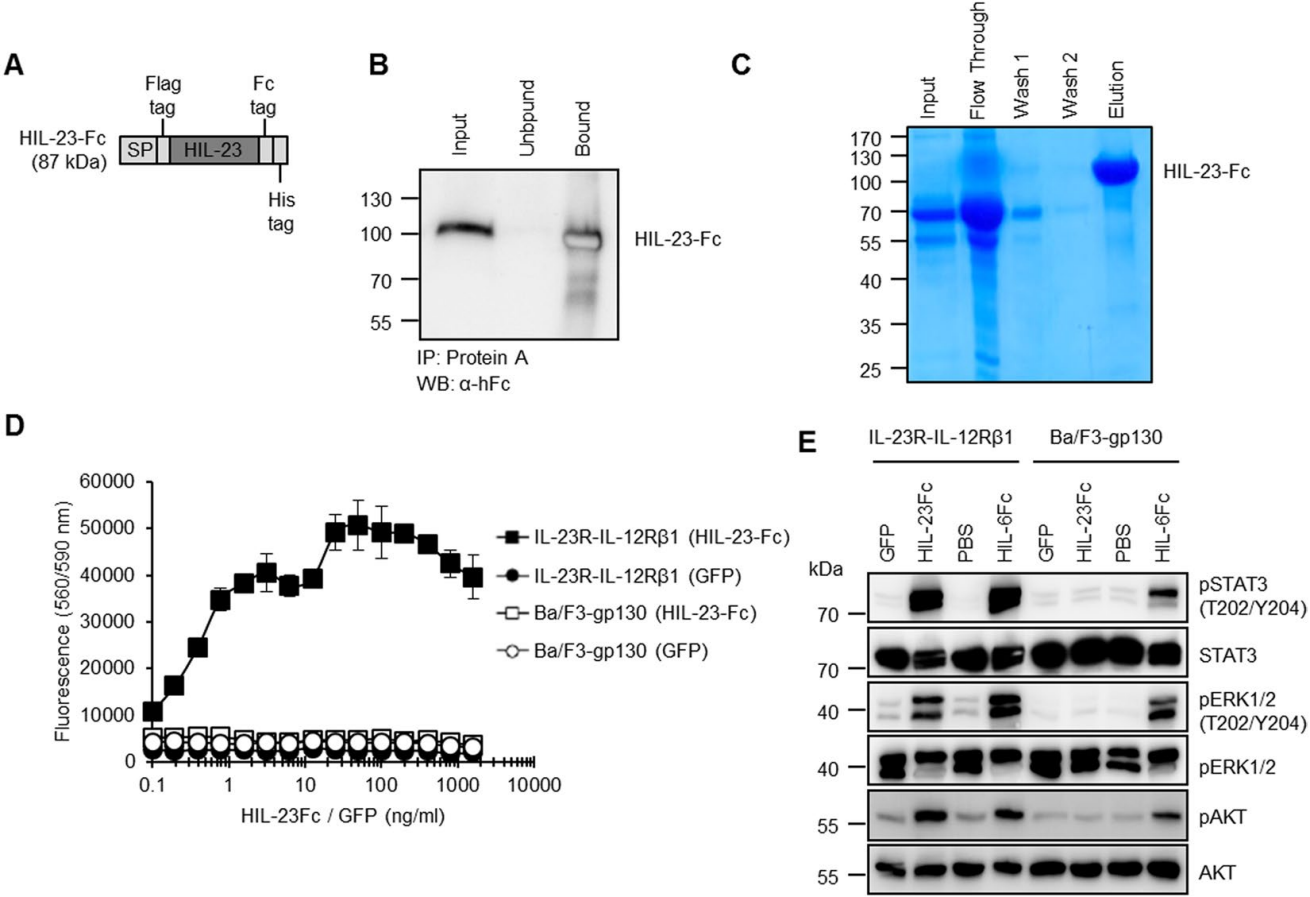
Biological activity of the purified recombinant HIL-23Fc. (**A**) Schematic illustration of the Hyper IL-23Fc fusion gene. Hyper-IL-23 was N-terminally tagged with a flag-tag and C-terminally tagged with Fc and His-tag. (**B**) CHO-K1 cells were stably transduced with Hyper-IL-23Fc cDNA and a high producer clone was selected. Western blot detection of HIL-23Fc was done with α-hFc mAbs. Input: CHO-K1 supernatant containing Hyper-IL-23Fc. Unbound: Detection of Hyper-IL-23Fc which was not bound to Protein A agarose after precipitation. Bound: Detection of Hyper-IL-23Fc, which was precipitated by Protein A agarose. (**C**) Hyper-IL-23Fc was purified by Protein A agarose affinity chromatography and stained by Coomassie brilliant blue. Input: cell culture supernatant from CHO-K1 cells stably expression Hyper-IL-23Fc. Flow Through: Fraction which did not bind to Protein A agarose. Wash: The column was washed twice with PBS. Elution: Hyper-IL-23Fc was eluted with 50 mM citrate buffer (50 mM citric acid, 50 mM sodium citrate), pH 3.25. (**D**) Ba/F3-gp130 and Ba/F3-mIL-23R-mIL-12Rβ1 cells were stimulated with the indicated concentrations of Hyper-IL-23Fc and GFP. Cellular proliferation was determined after 72 h. One representative experiment out of three is shown. Results are mean ± S.D. of three replicates. (**E**) Ba/F3-gp130 and Ba/F3-gp130-mIL-23R-mIL-12Rβ1 cells were stimulated with 25 ng/ml of recombinant GFP, HIL-23Fc, HIL-6Fc and PBS for 30 min. Equal amounts of total protein (50 μg/lane) were analyzed for phospho-STAT3, STAT3, phospho-ERK1/2, ERK1/2, phospho AKT and AKT. Western blot data show one representative experiment out of three. Uncropped images of Western blots are presented in Supplementary Figure 1.

To investigate potential therapeutic effects of IL-23 on myocardial remodeling after I/R we treated C57/BL6 wild-type mice with HIL-23Fc or PBS as control. Therfore we generated Hyper-IL-23Fc (HIL-23Fc), consisting of p40 and p19 connected by a flexible peptide linker, fused to a Fc-tag (Fig. 6A). For our studies we chose Hyper-IL-23Fc because it was shown that Hyper-cytokines are more active than the separate cytokine subunits^21^. In Hyper-cytokines, the cytokine subunits p19 and p40 are trapped in an active conformation by a flexible peptide linker. Moreover, the Fc-tag improved the stability of the protein and allowed a simple protein purification via ProteinA sepharose columns. To produce higher amounts of HIL-23Fc we first generated CHO-K1 cells that were stably expressing HIL-23Fc (Fig. 6B). Second, HIL-23Fc was purified from CHO-K1 cell culture supernatants via ProteinA affinity chromatography (Fig. 6C). Pure, recombinant HIL-23Fc was endotoxin-free confirmed by EndoZyme^®^ Detection Assay. Moreover, HIL-23Fc was highly biological active. As shown in Fig. 6D, half-maximal proliferation of IL-23-dependent Ba/F3-murine-IL-12Rβ1-murine-IL-23R cells was achieved with only 0.4 ng/ml HIL-23Fc, whereas no proliferation was inducible with green fluorescent protein (GFP) as control. HIL-23Fc-induced proliferation was specific, because Ba/F3-gp130 cells lacking the IL-23 receptor chains did not proliferate. Also analysis of signal transduction pathways in Ba/F3-mIL-12Rβ1/mIL-23R cells stimulated with HIL-23Fc demonstrated specificity and high activity of our recombinant Hyper IL-23Fc (Fig. 6E).

**Figure 7:**
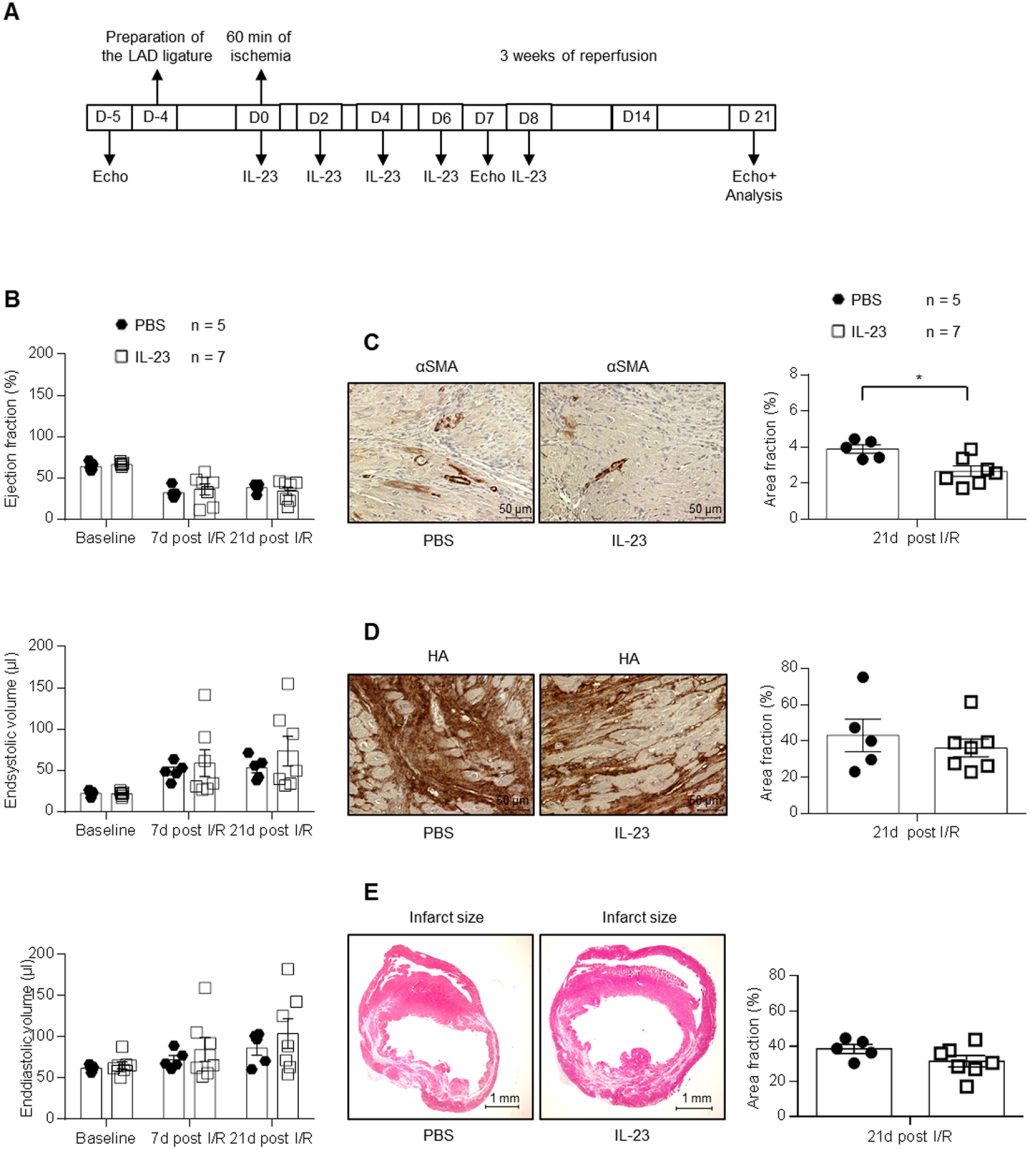
Closed-chest LAD ischemia/reperfusion with 60 min ischemia and 3 weeks of reperfusion was induced in wild-type mice treated with recombinant HIL-23Fc. (**A**) Schematic illustration of closed-chest LAD experiments with 12–14 weeks old wild-type mice injected with Hyper-IL-23Fc. Baseline measurement were performed at day -5, LAD ligature was inserted at day -4, 60 min of ischemia was done at day 0 followed by 3 weeks of reperfusion and analysis at day 1, 4 and 21. 10 µg/mouse HIL-23Fc in 200 µl sterile PBS or 200 µl sterile PBS were injected intraperitoneally on day 0 (directly at the beginning of reperfusion), 2, 4, 6 and 8. (**B**) Echocardiographic analysis of ejection fraction, endsystolic- and enddiastolic volume before (baseline) and after 7 d and 21 d of reperfusion. Results are mean ± S.E.M. of 5–7 animals/group (PBS treated group n = 5; HIL-23Fc treated group n = 7). (**C**) Immunostaining and quantitative analysis of αSMA protein in the LV after HIL-23Fc injection or PBS and after 3 weeks of reperfusion; 40x magnification; scale bars, 50 µm. Results are mean ± S.E.M. of 5–7 animals/group (PBS treated group n = 5; HIL-23Fc treated group n = 7). (**D**) Immunostaining with HAbP and quantitative analysis of HA amounts in the LV after HIL-23Fc injection or PBS and after 3 weeks of reperfusion. Representative images and quantitative analysis; 40x magnification; scale bars, 50 µm. Results are mean ± S.E.M. of 5–7 animals/group (PBS treated group n = 5; HIL-23Fc treated group n = 7). (**E**) Representative Gomori’s 1-step trichrome staining of heart sections and quantitative analysis of infarct size/LV after HIL-23Fc injection or PBS and after 3 weeks of reperfusion; scale bars, 1 mm. Results are mean ± S.E.M. of 5–7 animals/group (PBS treated group n = 5; HIL-23Fc treated group n = 7). Significance of difference (two-tailed Student t test): *p < 0.05.

Closed-chest LAD ischemia/reperfusion with 60 min ischemia and 3 weeks reperfusion was performed in C57/BL6 wild-type mice, which were treated with 10 µg/mouse HIL-23Fc diluted in sterile 200 µl PBS or as control injected with 200 µl sterile PBS intraperitoneally directly with onset of reperfusion and follow up on day 2, 4, 6 and 8. The left ventricle function was analyzed by echocardiography after 1 week and 3 weeks of I/R (Fig. 7A). No statistical differences in ejection fraction, endsystolic volume and enddiastolic volume after 1 week and 3 weeks of reperfusion were detected in the IL-23 and control group (Fig. 7B). To anylze the therapeutic effects of IL-23, mice were sacrificed and the ischemic hearts were taken 3 weeks after I/R and immunostaining of αSMA and HA was performed. The evaluations showed that in HIL-23Fc treated mice the amount of αSMA but not of HA was significantly decreased (Fig. 7C and D). Furthermore, we quantified the infarct sizes in the HIL-23Fc-treated group and control-group and found no changes between both groups (Fig. 7E). Our results showed that therapeutic treatment with IL-23 has no effects on the outcome of closed-chest LAD ischemia/reperfusion. Thus, we could not reproduce the negative effects of IL-23 on cardiac remodeling after I/R that was observed by two groups after IL-23 injection in rats^6,22^.

## Discussion

Taken together our study fails to reproduce the positive or negative effects of IL-23 on myocardial infarction despite using cutting-edge cardiovascular phenotyping by high resolution echocardiography and MRI. At this juncture, we can only speculate about the reasons, why we were not able to detect/reproduce a phenotype in IL-23R-Y416FΔICD signaling deficient mice after myocardial infarction. First of all, it became more and more clear that hygiene and housing conditions in the animal facilities have a major impact on the outcome of disease animal models and at least to some extent also on the clinical development success rates of novel drugs^23,24^. Second, we used 50–60 min of ischemia followed by reperfusion which is a more clinical relevant model of acute myocardial infarction as compared to permanent ligation because in MI patients coronary blood flow is usually restored. Permanent ligation was, however, applied in all other studies with IL-23 deficient mice^4–6,25^, but it causes also a milder disease formation with a limited loss of cardiac function, reduced cardiac remodeling and a less dynamic inflammatory response compared to ischemia/reperfusion models^26,27^.

Third, IL-23 biology is complex and deletion of IL-23p19 does not only affect IL-23 signaling but might also result in abrogation of IL-39^10^. Therefore deletion of the IL-23R which is the only signal-transducing receptor chain within the IL-23 receptor complex might have a different outcome as deletion of IL-23p19. Finally, IL-23 is a composite cytokine consisting of p19 and p40. p40 has at least two additional functions apart from its role as α-chain in IL-23. Together with p35, p40 forms also IL-12^9^. It might be possible that in the absence of p19, p40 might forms more IL-12. On the other hand, p40 has also immune-regulatory functions, most likely as a p40 homodimer. Interestingly, whereas IL-12 and IL-23 are pro-inflammatory cytokines, homodimeric p40 has anti-inflammatory function^28–30^. In the absence of p19, no pro-inflammatory IL-23 is produced but the surplus of free p40 might also contribute to phenotypes observed in IL-23p19 deficient mice. In conclusion, our data using the more clinically relevant LAD ischemia/reperfusion model demonstrated that IL-23 plays no role in myocardial infarction.

## Methods

### Animals and ethics statement

Specific pathogen-free IL-23R (wild-type), IL-23R-Y416 and Cre-recombined IL-23R-Y416FΔICD mice were obtained from the animal facility of the University of Duesseldorf. All mice were on C57BL/6 background. Mice were fed with a standard laboratory diet and given autoclaved tap water ad libitum. They were kept in an air-conditioned room with controlled temperature (20–24 °C), humidity (45–65%), and day/night cycle (12 h light, 12 h dark). Mice were acclimatized for at least 1 week before entering the study. All mouse experiments were performed according to the requirements of LANUV NRW with approval numbers: 84-02.04.2014.A114 and 84-02.04.2015.A489.

### Generation and genetic analysis of IL-23R-Y416F and IL-23R-Y416FΔICD mice

Floxed IL-23R-Y416F mice were generated by ingenious targeting laboratory (www.genetargeting.com). The conditional targeting vector bearing the point mutation was electroporated in C57BL/6N embryonic stem cells. Targeted embryonic stem cells were microinjected into Balb/c blastocysts and resulting chimeras with a high percentage of black coat color were mated to wild-type C57BL/6N mice to generate F1 heterozygous offspring. Confirmed F1 heterozygotes were set up for mating with C57BL/6N FLP mice to generate somatic Neo deleted mice. Heterozygous mice were confirmed by PCR and mated to wild-type C57BL/6N mice to generated homozygous floxed IL-23R-Y416F mice. These mice were crossed with mice expressing Cre-recombinase under a human cytomegalovirus (CMV) minimal promotor^31,32^. Human CMVCre^+/−^/IL-23R-Y416F^−/+^ mice were bred with IL-23R-Y416F mice. From the resulting offspring homozygous IL-23R-Y416F or homozygous IL-23R-Y416FΔICD mice were used for the experiments. DNA from tail clippings was isolated using the DirectPCR-Tail kit with proteinase K (Peqlab, Erlangen, Germany) following the manufactures instructions for genomic PCR. In detail, several PCRs were performed to confirm the introduction of the 3′loxP site after exon 11 (Primer P1/P2 - IL-23R: 322 bp; IL-23R-Y416F: 390 bp; IL-23R-Y416FΔICD: no PCR product), the 5′loxP/FRT sites (Primer P3/P4 - IL-23R: no PCR product; IL-23R-Y416F: 422 bp; IL-23R-Y416FΔICD: 422 bp), exon 11 (Primer P5/P6 - IL-23R: 223 bp; IL-23R-Y416F: 223 bp; IL-23R-Y416FΔICD: no PCR product), exon 10 (Primer P7/8 - IL-23R: 790 bp; IL-23R-Y416F: 790 bp; IL-23R-Y416FΔICD: 790 bp), and by multiplex PCR the presence or absence of exon 11 (Primer P3/P9 - IL-23R: 413 bp; P3/P9 - IL-23R-Y416F: 578 bp; P3/P2 - IL-23R-Y416FΔICD: 741 bp). To verify that the genetic modifications still resulted in expression of the IL-23R mRNA in IL-23R-Y416F and IL-23R-Y416FΔICD mice the primers RT1/RT2 located in exon 8 and exon 10 were used. Primer sequences are listed in Supplementary Table 1.

### Splenocyte culture

Spleen cells from 6 to 8-week-old IL-23R, IL-23R-Y416F and IL-23R-Y416FΔICD mice were passed through a nylon mash into a 50 ml tube containing PBS. The cell suspension was centrifuged at 300 × g for 5 min at 4 °C. Splenocytes were incubated for 5 days with 10 µg/ml anti-CD3 (cat. MAB484) and 5 µg/ml anti-CD28 (cat. AF483), in the absence or presence of 10 ng/ml human TGF-β1 (cat. 100-B), 40 ng/ml mouse IL-6 (cat. 406-ML) and 10 ng/ml mouse IL-1β (cat. 401-ML) (R&D Systems, Minneapolis, MN, USA) at 37 °C with 5% CO_2_. After 5 days, spleen cells were analyzed by flow cytometry and IL-17 concentrations in supernatants were determined by ELISA (cat. DY421, R&D Systems, Minneapolis, MN, USA).

### Cell surface detection of IL-23R in IL-23R-Y416F and IL-23R-Y416FΔICD spleen cells

To detect cell surface expression of IL-23R, activated spleen cells from IL-23R, IL-23R-Y416F and IL-23R-Y416FΔICD mice were washed with FACS buffer (PBS containing 1% BSA) and incubated with 5 × 10^5^ cells/100 μl FACS buffer supplemented with a 1:100 dilution of IL-23R antibody, (cat. 150903, Biolegend, San Diego, CA, USA) for 1 h on ice. Finally, cells were washed once with FACS buffer, suspended in 500 μl FACS buffer and analyzed by flow cytometry (BD FACSCanto II flow cytometer, BD Biosciences, San Jose, CA, USA). Data was evaluated using the FCS Express 4 Flow software (De Novo Software, Los Angeles, CA, USA).

### ELISA quantification of IL-17

ELISA for IL-17 (mouse IL-17 DuoSet, cat. DY421, R&D Systems, Minneapolis, MN, USA) were performed following the manufacturer’s instructions. Peroxidase reaction was stopped by adding 50 µl 1.8 N H_2_SO_4_. The absorbance was determined by the Tecan Infinite 200pro fluorometer (Tecan, Crailsheim, Germany).

### Closed and open-chest model of Ischemia/reperfusion injury

Ischemia followed by reperfusion was induced by closed-chest and open-chest model as described elsewhere^33,34^. For closed-chest, 12–14 week old male mice were anesthetized by intraperitoneal injection of ketamine (60 mg/kg BW) and xylazine (10 mg/kg BW), intubated and connected to a rodent ventilator (Minivent Microventilator, Hugo Sachs, Germany). Body temperature was maintained at 37 °C via a heating plate. After precordial left lateral thoracotomy and pericardectomy, the LAD was identified and tunneled using a 0–7 prolene thread. After chest closure the ends of the thread were placed inside a subcutaneous pocket before closing of the skin and injection of buprenorphine (0.05–0.1 mg/kg, s.c. every 8 h). At postoperative day 4, mice were anesthetized by inhalation of isoflurane 2.0 Vol%, the subcutaneous thread was retrieved and the LAD was occluded by cautiously pulling both ends apart. Continuous electrocardiogram (ECG) monitoring of ST elevations confirmed myocardial ischemia. After 60 min occlusion, tension was relieved, reperfusion was verified by ECG changes, and the thread was cut off at the chest wall before wound closure and injection of buprenorphine (0.05–0.1 mg/kg, s.c. every 8 h). Mice were sacrificed, serum was collected and hearts were harvested after 24 h, 4 days or 3 weeks of reperfusion.

For open-chest also 12–14 week old male mice were intubated and anesthetized by mechanical ventilation with isoflurane (2.0%) at a rate of 150 strokes/min and a tidal volume of 250–300 μl. Each animal was placed in a supine position with paws taped to an ECG board (leadII) to measure ST segment elevations during myocardial infarction. The chest was then opened with a lateral cut along the left side of the sternum. Subsequently, the pericardium was gently dissected to allow visualization of coronary artery anatomy. Ligation was proceeded with an 8-0 polypropylene suture with a tapered needle passed underneath the LAD, 1–3 mm from the tip of the left auricle. The success of infarction was verified microscopically by the absence of blood flow in the epicardium as well as significant elevations of ST segment. After 50 min, the snare occluder was opened to initiate reperfusion. The chest was then closed with 6-0 polypropylene suture with one layer through the muscle and a second layer through the skin and subcutaneous material. For analgesia, buprenorphine is administered half an hour before surgery and postoperatively (0.05–0.1 mg/kg, s.c. every 8 h up to 5 d). Mice were sacrificed and hearts were harvested after 4 weeks of reperfusion.

### Echocardiography

For characterization of left ventricular (LV) function a Vevo 3100 High-Resolution *In Vivo* Micro-Imaging System and a 30-MHz scanhead (VisualSonics Inc., Toronto, Canada) was used. Echocardiography was performed as previously described^35^. LV end-systolic volumes and end-diastolic volumes were measured and the ejection fractions were calculated.

### PFC injection

Mice were anesthetized with isoflurane (2.0%) using a home-built nose cone. A dosage of 0.6 mM/kg (for fluorescence experiments) or up to 3 mM/kg body weight (for MRI) of the PFC emulsion was given intravenously after 24 h of reperfusion.

### Magnetic resonance imaging

For cardiac analysis, data were recorded on a Bruker Avance III 9.4-T widebore (89 mm) NMR spectrometer operating at frequencies of 400.13 MHz for ^1^H and 376.46 MHz for ^19^F measurements as described^36,37^. Experiments were performed using a Bruker microimaging unit (Micro 2.5) equipped with an actively shielded 40-mm gradient set (capable of 1500 mT/m maximum gradient strength and 110 μs rise time at 100% gradient switching, a 25-mm ^1^H/^19^F bird cage resonator and Paravision 5.1 as operating software). Mice were anesthetized with 1.5% isoflurane in a water-saturated gas mixture of 20% oxygen in nitrogen applied at a rate of 75 ml/min by manually restraining the animal and placing its head in a home-build nose cone. The frontpaws and the left hindpaw were attached to ECG electrodes (Klear-Trace, CAS Medical Systems, Branford). Respiration was monitored from a pneumatic pillow positioned at the animal’s back. Vital functions were acquired by a M1025 system (SA Instruments, Stony Brook) and used to synchronize data acquisition with cardiac and respiratory motion. Throughout the experiment, mice were respiring spontaneously at a rate of approximately 100 min^−1^ and were kept at 37 °C.

### Cardiac imaging

For functional analysis ^1^H images of murine hearts were acquired using an ECG- and respiratory-triggered fast gradient echo cine sequence essentially as described^36,37^. A flip angle of 15°, echo time (TE) of 1.8 ms, and a repetition time (TR) of about 4 ms were used. The pixel size after zero filling was 117 × 117 μm^2^ (field of view (FOV) 30 × 30 mm^2^, matrix 128 × 128, slice thickness (ST) 1 mm, acquisition time per slice for one cine sequence 1–2 min). For late gadolinium enhancement (LGE), a bolus of Gd-DTPA (0.2 mmol Gd-DTPA per kg body weight) was applied intraperitonally. ^19^F images were recorded using a multislice RARE sequence (8 slices, RARE factor 64, FOV 30 × 30 mm^2^, matrix 64 × 64 resulting in a pixel size after zero filling of 234 × 234 μm^2^, ST 1 mm, TR 4.5 s, TE 3.38 ms, 256 averages, acquisition time, 19.12 min). No cardiac or respiratory gating was applied for ^19^F MRI. For fusion with ^19^F images additional ^1^H datasets with a ST of 2 mm were recorded. The full experimental protocol for cardiac MR studies, respectively, including both ^1^H and ^19^F imaging took around 1 h and was well tolerated by all mice which recovered from anesthesia within 1–2 min after removal of the nose cone. For evaluation of functional parameters (EDV, ESV and EF), ventricular demarcations in end-diastole and end-systole were manually drawn in all slices with the ParaVision region-of-interest tool (Bruker, Rheinstetten, Germany).

### Volumetric analysis of inflamed areas

Anatomical matching multislice ^1^H and ^19^F MR data sets were used to quantify inflamed myocardial tissue^37^. Affected volumes were calculated from ^19^F images by planimetric analysis of PFC signals, multiplication with the slice thickness, and summation over all slices. In order to correct for differences in organ size obtained values were related to total heart volumes, which were assessed from the corresponding ^1^H images. ^19^F signal-to-noise ratios were determined from PFC signals originating from the myocardium only, and were averaged over the entire region containing PFCs. Signals derived from liver, chest, skull, lymph nodes or other tissues were not included into the calculation.

### Serum biochemistry

Troponin T was measured by Universitätsklinikum Düsseldorf, Institute of Clinical Chemistry and Laboratory Diagnostics.

### Histochemistry

Affinity histochemistry of hyaluronic acid (HA) was performed with biotinylated bovine HAbP (1:250, cat. 385911-50UG, Merck, Darmstadt, Germany)^38^. Galectin-3 staining was performed using the anti-MAC2-antibody (1:200, cat. CL894AP, Cedarlane, Burlington, USA) and alpha-smooth muscle actin (αSMA) was detected with anti-αSMA antibody (1:200, cat. ab5649, Abcam, Cambridge, UK). Streptavidin Peroxidase (1:200, cat. S5512-1MG, Sigma Aldrich, Munich, Germany), horseradish peroxidase (HRP)-conjugated secondary antibodies (1:600 cat. sc-2006 and 1:200 cat. sc-2004, Santa Cruz, Dallas, USA) and 3,30-diaminobenzidine (DAB) (cat. DAB530, Zytomed, Berlin, Germany) were used for visualization. For the quantification of HA, Galectin-3 and αSMA ImageJ software (NIH, Bethesda, MD) was used.

### Analysis of infarct size

Three and four weeks of reperfusion, animals were sacrificed and hearts were explanted for infarct size measurement. Formalin-fixed hearts were sliced in serial sections (level 1–5) and stained with Gomori’s 1-step trichrome staining (Sigma Aldrich, Munich, Germany). For the quantification of infarct size in the left ventricle, 15 images of each were taken with the microscope (Keyence BZ-9000, Keyence Deutschland GmbH, Neu-Isenburg, Germany) from levels 2–5 in a 40-fold magnification and were quantified with ImageJ software (NIH, Bethesda, MD).

### Gene expression analysis

RNA was isolated with TRIzol (Thermo Fisher Scientific, Waltham, MA, USA) according to the manufacturer’s instructions. 5 µg RNA was used for cDNA synthesis using RevertAid reverse transcriptase (Thermo Fisher Scientific, Waltham, MA, USA) following manufacturer’s instructions with standard oligo-dT method. Using SYBR^®^Green PCR Master Mix (Thermo Fisher Scientific, Waltham, MA, USA), the qPCR reaction was performed in triplicates using 25 ng of the cDNA as a template. The fluorescence detection and measurements were taken using ABI 7500 Real-Time PCR System (Applied Biosystems (Thermo Fisher Scientific, Waltham, MA, USA)). GAPDH was chosen as endogenous control. The primer pair’s sequences are listed in Supplementary Table 2.

### Expression and purification of HIL-23Fc

Cloning of HIL-23Fc was described previously^39^. CHO-K1 cells (ACC-110, Leibniz Institute DSMZ-German Collection of Microorganisms and Cell Cultures, Braunschweig, Germany) were transfected with TurboFect (Fermentas, Thermo Scientific). 48 h after transfection cells were split into fresh medium containing 1.125 mg/ml Geneticin^®^ (Genaxxon Bioscience GmbH, Ulm, Germany). Cells were fed with selective medium every 3–4 days until Geneticin^®^-resistant foci were identified. Single colonies were picked, expanded in 96-well plates and analyzed by Western blot. High expressing clones were chosen for a second round of Geneticin^®^ selection to establish a stable CHO-K1 cell line. Cells were expanded and cultured in high glucose culture medium supplemented with 5% low ultra-low IgG fetal bovine serum (Gibco, Life Technologies, Darmstadt, Germany), 60 mg/liter penicillin, and 100 mg/liter streptomycin (Genaxxon Bioscience GmbH, Ulm, Germany) at 37 °C with 5% CO_2_. Supernatant was harvested by two-step centrifugation (15 min, 1000 × g, 15 min 10000 × g, 4 °C) and filtered through a 0.45 µm bottle top. HIL-23Fc was purified by Protein A agarose affinity chromatography. Hyper-IL-23Fc was eluted with 50 mM citrate buffer (50 mM citric acid, 50 mM sodium citrate), pH 3.25. HIL-23Fc was tested for endotoxins with EndoZyme^®^ Detection Assay (Hyglos GmbH, Bernried, Germany). The concentration of HIL-23Fc protein was determined by ELISA (Mouse IL-12/IL-23 p40 DuoSet, R&D Systems, Minneapolis, MN, USA) following the manufacturer’s instructions.

### Coomassie Brilliant Blue Staining

SDS gels were incubated for 1 h in Coomassie Brilliant Blue stain (water supplemented with 0.1% Coomassie Brilliant Blue R250, 10% acetic acid, and 40% methanol) under gentle agitation. Coomassie Brilliant Blue stain was replaced by destaining solution (water supplemented with 20% methanol and 10% acetic acid) and kept under gentle agitation.

### Immunoprecipitation

Cell culture supernatant from stable CHO-K1 cell line expressing HIL-23Fc was incubated with 50 µl of Protein A agarose beads (Roche Diagnostics, Mannheim, Germany) at 4 °C for 4 h under gentle agitation. Immunoprecipitates were washed three times with PBS. Afterwards 50 μl of Laemmli sample buffer (500 mm Tris-HCl, pH 6.8, 2% (w/v) SDS, 2% (w/v) 2- mercaptoethanol, 20% (v/v) glycerol and 0.03% bromphenol blue) were added to Protein A agarose beads and incubated at 95 °C for 10 min. Interaction was analyzed by Western blotting using α-Fc antibodies (1:1000, cat. #31423, rabbit anti-human IgG, Fcγ fragment specific, peroxidase conjugated, Pierce, Thermo Scientific, Rockford, IL, USA).

### Cell viability assays of murine Ba/F3 cells

Murine Ba/F3-gp130 cells were obtained from Immunex (Seattle, WA, USA)^40^. Ba/F3-mIL-23R-mIL-12Rβ1 cells were described previously^39^. All Ba/F3 cell lines were grown in DMEM high glucose culture medium (GIBCO^®^, Life Technologies, Darmstadt, Germany) supplemented with 10% fetal bovine serum (GIBCO^®^, Life Technologies), 60 mg/l penicillin and 100 mg/l streptomycin (Genaxxon bioscience GmbH, Ulm, Germany) at 37 °C with 5% CO_2_. Ba/F3-gp130 cells or variants thereof were maintained in the presence of Hyper-IL-6 (HIL-6), a fusion protein of IL-6 and the soluble IL-6R^41^. To remove the cytokines, Ba/F3 cell lines were washed three times with sterile PBS. In all, 5 × 10^3^ cells were suspended in DMEM supplemented with 10% FCS, 60 mg/l penicillin and 100 mg/l streptomycin and cultured for 3 days in a final volume of 100 μl with or without recombinant proteins as indicated. The CellTiter-Blue Cell Viability Assay (Promega, Karlsruhe, Germany) was used to estimate the number of viable cells by recording the fluorescence (excitation 560 nm, emission 590 nm) using the Infinite M200 PRO plate reader (Tecan, Crailsheim, Germany) immediately after adding 20 μl of reagent per well (time point 0) and up to 2 h after incubation under standard cell culture conditions. All of the values were measured in triplicate per experiment. Fluorescence values were normalized by subtraction of time point 0 values.

### Stimulation assays and Western blotting

For analysis of STAT3 and ERK1/2 and AKT Ba/F3-gp130 cell lines with and without expression of murine IL-23R/IL-12Rβ1 were washed three times with sterile PBS and incubated in serum-free DMEM for at least 4 h. Cells were stimulated with 25 ng/ml recombinant GFP, HIL-23Fc, HIL-6Fc or PBS as control. Protein concentration of cell lysates was determined by BCA Protein Assay (Pierce, Thermo Fisher Scientific,Waltham, MA, USA). Analysis of STAT3 and ERK1/2 and AKT activation was done by immunoblotting using 50 μg total protein from total cell lysates, separated by sodium dodecyl sulfate polyacrylamide gel electrophoresis under reducing conditions and transferred to polyvinylidene fluoride (PVDF) membranes (Carl Roth, Karlsruhe, Germany). The membranes were blocked in 5% fat-free dried skimmed milk (Carl Roth, Karlsruhe, Germany) in TBS-T (10 mM Tris-HCl (Carl Roth, Karlsruhe, Germany) pH 7.6, 150 mM NaCl (AppliChem, Darmstadt, Germany), 0.5% Tween 20 (Sigma Aldrich, Munich, Germany)) and probed with the indicated primary antibodies in 5% fat-free dried skimmed milk in TBS-T (STAT3 (124H6) (1:1000, cat. #9139), or 5% BSA (Carl Roth, Karlsruhe, Germany) in TBS-T (phospho-STAT3 (Tyr705) (D3A7) (1:1000, cat. #9145), phospho-ERK1/2 (Thr-202/Tyr-204) (D13.14.4E) (1:1000, cat. #4370), ERK1/2 (1:1000, cat. #9102), phospho-AKT (Ser473) (1:1000, cat. #9271), AKT (1:1000, cat. #9272)), at 4 °C overnight. After washing, the membranes were incubated with secondary peroxidase-conjugated antibodies (Thermo Fisher Scientific, Waltham, MA, USA, cat. 31462, cat. 31451) 1:5000 diluted in 5% fat-free dried skimmed milk in TBS-T for 1 h at room temperature. PageRuler Prestained Protein Ladder (Thermo Fisher Scientific, Waltham, MA, USA, cat. #26616) was used as MW Marker. The Immobilon™ Western Chemiluminescent HRP Substrate (Merck Chemicals GmbH, Darmstadt, Germany) and the ChemoCam Imager (INTAS Science Imaging Instruments GmbH, Göttingen, Germany) were used for signal detection. For re-probing with another primary antibody, the membranes were stripped in 62.5 mM Tris-HCl (Carl Roth, Karlsruhe, Germany) pH 6.8, 2% SDS (Carl Roth, Karlsruhe, Germany) and 0.1% β-mercaptoethanol (Sigma Aldrich, Munich, Germany) for 30 min at 60 °C and blocked again.

### Statistical analysis

Data are presented as mean ± S.E.M. or ± S.D. For multiple comparisons, one-way ANOVA, followed by Bonferroni post hoc tests, was used (GraphPad Prism 6.0, GraphPad Software Inc., San Diego, CA, USA). Two-group comparisons were performed with two-tailed Student t test. Statistical significance was set at the level of p < 0.05.

## Electronic supplementary material

Dataset 1

## Data Availability

The authors declare that the data supporting the findings of this study are available within the paper and from the authors on request.

## References

[1] Marchant D (2012). Inflammation in myocardial diseases. Circulation research.

[2] Ridker P (2017). Antiinflammatory Therapy with Canakinumab for Atherosclerotic Disease. N Engl J Med.

[3] Fontes J, Rose N, Čiháková D (2015). The varying faces of IL-6: From cardiac protection to cardiac failure. Cytokine.

[4] Savvatis K (2014). Interleukin-23 deficiency leads to impaired wound healing and adverse prognosis after myocardial infarction. Circulation: Heart Failure.

[5] Yan X (2012). Deleterious effect of the IL-23/IL-17A axis and γδT cells on left ventricular remodeling after myocardial infarction. J Am Heart Assoc.

[6] Hu X (2016). IL-23 Promotes Myocardial I/R Injury by Increasing the Inflammatory Responses and Oxidative Stress Reactions. Cellular Physiology and Biochemistry.

[7] Sommer J (2012). Constitutively Active Mutant gp130 Receptor Protein from Inflammatory Hepatocellular Adenoma Is Inhibited by an Anti-gp130 Antibody That Specifically Neutralizes Interleukin 11 Signaling. J Biol Chem.

[8] Wang X (2016). A novel IL-23p19/Ebi3 (IL-39) cytokine mediates inflammation in Lupus-like mice. European Journal of Immunology.

[9] Collison Lauren W., Vignali Dario A. A. (2008). Interleukin-35: odd one out or part of the family?. Immunological Reviews.

[10] Hasegawa H (2016). Expanding Diversity in Molecular Structures and Functions of the IL-6/IL-12 Heterodimeric Cytokine Family. Frontiers in Immunology.

[11] Collison LW, Vignali DA (2008). Interleukin-35: odd one out or part of the family?. Immunol Rev.

[12] McGeachy M (2009). The interleukin 23 receptor is essential for the terminal differentiation of interleukin 17-producing effector T helper cells *in vivo*. Nat Immunol.

[13] Cua DJ (2003). Interleukin-23 rather than interleukin-12 is the critical cytokine for autoimmune inflammation of the brain. Nature.

[14] Murphy C (2003). Divergent pro- and antiinflammatory roles for IL-23 and IL-12 in joint autoimmune inflammation. J Exp Med.

[15] Hue S (2006). Interleukin-23 drives innate and T cell-mediated intestinal inflammation. J Exp Med.

[16] Kullberg M (2006). IL-23 plays a key role in Helicobacter hepaticus-induced T cell-dependent colitis. J Exp Med.

[17] Awasthi A (2009). Cutting edge: IL-23 receptor gfp reporter mice reveal distinct populations of IL-17-producing cells. J Immunol.

[18] Buonocore S (2010). Innate lymphoid cells drive interleukin-23-dependent innate intestinal pathology. Nature.

[19] Floss D (2013). Identification of canonical tyrosine-dependent and non-canonical tyrosine-independent STAT3 activation sites in the intracellular domain of the interleukin 23receptor. J Bio Chem.

[20] Burkett PR (2015). Meyer zu Horste, G. & Kuchroo, V. K. Pouring fuel on the fire: Th17 cells, the environment, and autoimmunity. The Journal of Clinical Investigation.

[21] Rose-John S (1990). Studies on the structure and regulation of the human hepatic interleukin-6 receptor. European journal of biochemistry.

[22] Liao Y (2017). Promoting effects of IL-23 on myocardial ischemia and reperfusion are associated with increased expression of IL-17A and upregulation of the JAK2-STAT3 signaling pathway. Molecular Medicine Reports.

[23] Willyard C (2018). Squeaky clean mice could be ruining research. Nature.

[24] Hay M, Thomas D, Craighead J, Economides C, Rosenthal J (2014). Clinical development success rates for investigational drugs. Nat Biotechnol..

[25] Liao Y (2017). Promoting effects of IL-23 on myocardial ischemia and reperfusion are associated with increased expression of IL-17A and upregulation of the JAK2-STAT3 signaling pathway. Mol Med Rep.

[26] van Zuylen V (2015). Myocardial infarction models in NOD/Scid mice for cell therapy research: permanent ischemia vs ischemia-reperfusion. Springerplus.

[27] Vandervelde S (2006). Increased inflammatory response and neovascularization in reperfused vs. non-reperfused murine myocardial infarction. Cardiovasc Pathol.

[28] Shimozato O (2006). The secreted form of the p40 subunit of interleukin (IL)-12 inhibits IL-23 functions and abrogates IL-23-mediated antitumour effects. Immunology.

[29] Ling P (1995). Human IL-12 p40 homodimer binds to the IL-12 receptor but does not mediate biologic activity. J Immunol.

[30] Gillessen S (1995). Mouse interleukin-12 (IL-12) p40 homodimer: a potent IL-12 antagonist. European Journal of Immunology.

[31] Kühn R, Schwenk F, Aguet M, Rajewsky K (1995). Inducible gene targeting in mice. Science.

[32] Schwenk F, Baron U, Rajewsky K (1995). A cre-transgenic mouse strain for the ubiquitous deletion of loxP-flanked gene segments including deletion in germ cells. Nucleic Acids Res.

[33] Gorressen S (2015). Circulating NOS3 modulates left ventricular remodeling following reperfused myocardial infarction. PLoS One.

[34] Nossuli T (2000). A chronic mouse model of myocardial ischemia-reperfusion: essential in cytokine studies. Am J Physiol Heart Circ Physiol.

[35] Krusche C (2011). Desmoglein 2 mutant mice develop cardiac fibrosis and dilation. Basic research in cardiology.

[36] Flögel U (2005). Lack of myoglobin causes a switch in cardiac substrate selection. Circulation research.

[37] Flögel U (2008). *In vivo* monitoring of inflammation after cardiac and cerebral ischemia by fluorine magnetic resonance imaging. Circulation.

[38] Röck K (2012). Estradiol protects dermal hyaluronan/versican matrix during photoaging by release of epidermal growth factor from keratinocytes. J Bio Chem.

[39] Hummel T (2017). Synthetic Deletion of the Interleukin 23 Receptor (IL-23R) Stalk Region Led to Autonomous IL-23R Homodimerization and Activation. Molecular and cellular biology.

[40] Gearing AJ (1994). Processing of tumour necrosis factor-alpha precursor by metalloproteinases. Nature.

[41] Fischer M (1997). A bioactive designer cytokine for human hematopoietic progenitor cell expansion. Nat Biotechnol..

