# Supplementary material for: IL-23R Signaling Plays No Role in Myocardial Infarction
Source: Sci Rep. 2018 Nov 20;8:17078. doi: 10.1038/s41598-018-35188-8 (PMC6244091; doi:10.1038/s41598-018-35188-8)
Supplement: Supplementary file 1 — Dataset 1 [file 41598_2018_35188_MOESM1_ESM.pdf]

IL-23R SIGNALING PLAYS NO ROLE IN MYOCARDIAL INFARCTION

Erika Engelowski[1], Nastaran Fazel Modares[1], Simone Gorressen[2], Pascal Bouvain[3], Dominik Semmler[2], Christina Alter[3], Zhaoping Ding[3], Ulrich Flögel[3], Jürgen Schrader[3], Haifeng Xu[4], Philipp A. Lang[4], Jens Fischer[2], Doreen M. Floss[1], Jürgen Scheller[1, §]

[1]Institute of Biochemistry and Molecular Biology II, Medical Faculty, Heinrich-Heine University, 40225 Düsseldorf, Germany
[2]Institute of Pharmacology and Clinical Pharmacology, Medical Faculty, Heinrich-Heine University, 40225 Düsseldorf, Germany
[3]Institute for Molecular Cardiology, Medical Faculty, Heinrich-Heine University, 40225 Düsseldorf, Germany
[4]Institute of Molecular Medicine II, Medical Faculty, Heinrich-Heine-University, Düsseldorf, Germany

[§]Correspondence should be addressed to JS (email: jscheller@uni-duesseldorf.de, fax: +49 2 11-8 11 27 26)

SUPPLEMENTARY FIGURE LEGENDS

Supplementary Figure 1. Uncropped images of western blots presented in the main manuscript. Black or white dotted box indicates areas that were cropped.

Supplementary Table 1. List of PCR primer sequences.

Supplementary Table 2. List of Real-time qRT-PCR primer sequences.

Supplementary Figure 1

Fig 1D

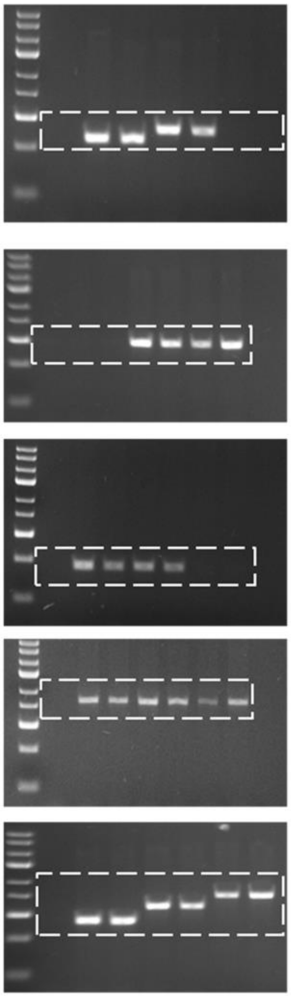

Fig 1E

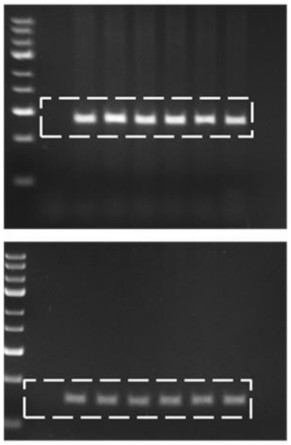

Fig 6C

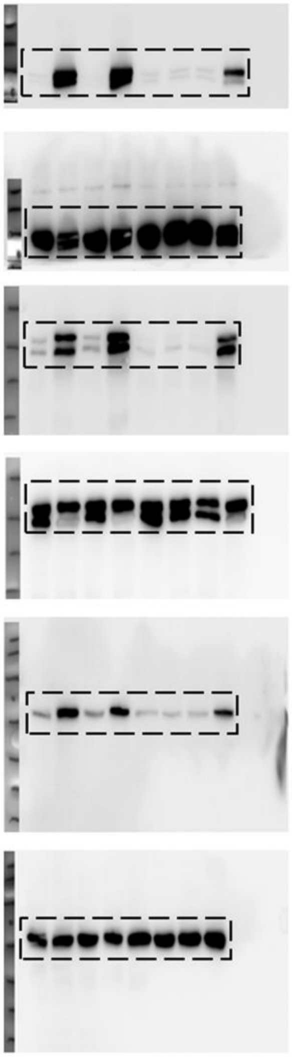

Supplementary Table 1

RT-PCR primers		
**Gene**	**Forward primer**	**Reverse primer**
Gapdh	5’-GAAGGGCTCATGACCACAGT-3’	5’-CATTGTCATACCAGGAAATGAGCT-3’
IL-23R	5’-AGCAAAATCATCCCACGAAC-3’	5’-GCCACTTTGGGATCATCAGT-3’
**PCR primers**		
**Name**	**Sequence**	
P1	5’-ACCCATGGGCTTCCTTTCTCTACT-3’	
P2	5’-CAGAGTTTCCATTAGGTGGTAAGC-3’	
P3	5’-GACCAGACCTATTTATGTGGCTCT-3’	
P4	5’-GGAACTTCGCTAGACTAGTACGCGTG-3’	
P5	5’-GAAAGAGACCCTACATCCCTTGA-3’	
P6	5’-CAGGAGACAAATTCATCAGACAAC-3’	
P7	5’-GCTAGAGGCCTCATAGGCTAAGTTGTACTCC-3’	
P8	5’-GTCCGCTGCAAGAGCAACAG-3’	
P9	5’-TGATGTGTATCTAGGCTACATCG-3’	

Supplementary Table 2

**Real-time qRT-PCR primers**		
**Gene**	**Forward primer**	**Reverse primer**
Gapdh	5’-GAAGGGCTCATGACCACAGT-3’	5’-CCTTCTTGATGTCATCATATTTGG-3’
RORγt	5’-TGCAAGACTCATCGACAAGG-3’	5’-AGGGGATTCAACATCAGTGC-3’
IFNγ	5’-ACTGGCAAAAGGATGGTGAC-3’	5’-ACCTGTGGGTTGTTGACCTC-3’
IL-17α	5’-CCTCAGACTACCTCAACCGTTC-3	5’-TTCATGTGGTGGTCCAGCTTTC-3
Col1A1	5’-ACGCCATCAAGGTCTACTGC-3	5’-ACTCGAACGGGAATCCATCG-3
Col3A1	5’-CAACCAGTGCAAGTGACCAA-3	5’-TGACCTGTATTGGGTGGTTG-3
Acta2	5’-CTGACAGAGGCACCACTGAA-3	5’-CATCTCCAGAGTCCAGCACA-3
CyclinD1	5’-GCGTACCCTGACACCAATCTC-3	5’-CTCCTCTTCGCACTTCTGCTC-3
Bcl2	5’-ATGCCTTTGTGGAACTATATGGC-3	5’-GGTATGCACCCAGAGTGATGC-3
Bcl2l1	5’-GATTCCCATGGCAGCAGGAAGCAAG-3	5’-CTCCATCCCGAAAGAGTTCATTCACT-3
